# Friend or foe: Face masks in patients with COPD at high altitude

**DOI:** 10.14814/phy2.71010

**Published:** 2026-07-23

**Authors:** Javier Josue Flores‐Mamani, Andree Ingala‐Tapia, Armin Alvaro Quispe‐Cornejo

**Affiliations:** ^1^ Instituto Académico Científico Quispe‐Cornejo La Paz Bolivia; ^2^ Medical School Universidad Mayor de San Andrés La Paz Bolivia; ^3^ Department of Intensive Care Erasme University Hospital, Université Libre de Bruxelles Bruxelles Belgium; ^4^ Present address: Medical School Universidad Mayor de San Andrés La Paz Bolivia

**Keywords:** arterial blood gases, chronic obstructive pulmonary disease, face mask, FFP2 mask, high‐altitude, N95 mask

## Abstract

High‐altitude environments impose unique physiological challenges due to reduced barometric pressure and lower inspired oxygen partial pressure, raising concerns about the safety of face mask use in individuals with chronic obstructive pulmonary disease. While evidence at sea level shows that surgical and N95 masks modestly impair gas exchange—slightly reducing oxygen uptake, oxygen saturation—they consistently increase perceived exertion, dyspnea, and fatigue. Limited studies conducted under hypobaric conditions—typically involving small sample sizes, brief mask‐wearing periods, and healthy participants. However, data at higher altitudes and in patients with COPD remain scarce. Emerging epidemiological evidence suggests that COPD prevalence may decrease with increasing altitude, yet high‐altitude residence can alter pathophysiology and could increase vulnerability to hypoxemia and hypercapnia during mask use in advanced disease. Existing studies in COPD at sea level report generally modest physiological effects with short‐term mask use, but their small sample sizes and short exposure durations limit generalizability. This perspective highlights the urgent need for well‐designed studies evaluating both acute and chronic physiological responses to prolonged mask use in COPD patients at high altitudes to inform evidence‐based public health recommendations.

## 1 INTRODUCTION

Face masks are widely used in healthcare settings and, increasingly, by the general population as a measure to prevent the transmission of respiratory diseases (Li et al., 2022). The COVID‐19 pandemic expanded this practice globally, establishing mask use as a standard intervention to reduce SARS‐CoV‐2 transmission; nevertheless, respiratory illnesses continue to represent a substantial burden on global public health (World Health Organization, 2024). In parallel, atmospheric pollution also plays an important role in human health, and the World Health Organization (WHO), European Region, encourages the use of respirators such as N95 or KN95 as part of individual‐level actions to reduce exposure to this environmental hazard (World Health Organization, 2026). Consequently, face mask use serves functions ranging from the prevention of infectious respiratory diseases to protection against exposure to atmospheric pollution.

However, the use of face masks under high‐altitude conditions raises specific physiological concerns. Altitude is classically divided into five categories: intermediate (1500–2500 m), high (2500–3500 m), very high (3500–5800 m), extreme (>5800 m), and the so‐called “death zone” (>8000 m) (Imray et al., 2011). As elevation increases, barometric pressure decreases, leading to a progressive reduction in inspired and arterial oxygen partial pressure (PIO_2_ and PaO_2_, respectively) (Grocott et al., 2009; Imray et al., 2011). Under these conditions, even small decreases in PaO_2_ along the oxyhemoglobin dissociation curve may result in disproportionately large declines in arterial oxygen saturation (SaO_2_) compared with sea level, particularly during prolonged exposure or additional respiratory loads such as those imposed by face masks (Baylis & Till, 2009). Mask use may therefore further reduce SaO_2_ at high altitude, potentially exacerbating underlying respiratory impairments, including chronic obstructive pulmonary disease (COPD), in individuals with limited ventilatory reserve.

This issue is especially relevant given that approximately 500.3 million people worldwide live above 1500 m, including 81.6 million at elevations ≥2500 m and 14.4 million at ≥3500 m (Tremblay & Ainslie, 2021). Notable high‐altitude populations include residents of El Alto, Bolivia (4150 m), one of the highest major cities in the world; Lhasa, China (3700 m), the cultural capital of Tibet; and Breckenridge, Colorado, USA (2925 m), a prominent high‐altitude resort town (CDC, 2025). In this context, determining whether face mask use represents a protective strategy or a physiological burden for patients with COPD living at high to very high altitudes remains an important and insufficiently explored question. This perspective aims to provide practical insights to guide future research on the effects of face mask use in adults with COPD residing at high‐altitude environments.

## 2 HIGH ALTITUDE PHYSIOLOGY

When people from sea level rapidly ascend to altitude, acclimatization starts to cope with hypoxia (West et al., 2012). A key molecular mechanism involves the increased expression of Hypoxia‐Inducible Factor (HIF), which boosts genes that regulate physiological processes for acclimatization (Luks & Hackett, 2022). The initial response consists of a chemoreceptor‐mediated increase in ventilatory drive, which elevates tidal volume (VT) and respiratory rate (RR). This respiratory stimulation is accompanied by heightened sympathetic outflow, resulting in increased heart rate (HR) and cardiac output (CO) (Zidan et al., 2025). However, ventricular volumes are maintained; later, left ventricular filling and stroke volume are lowered because of pulmonary arterial pressure (PAP) increases due to hypoxic pulmonary vasoconstriction (Williams et al., 2022) mediated by HIF that induces major production of endothelin‐1 (West et al., 2012). In the brain, the decreases of arterial oxygen content (CaO_2_) triggers an increase in blood flow to ensure adequate perfusion and maintain adequate delivery of oxygen and nutrients to keep normal brain function, although the degree of acclimatization varies among individuals (Luks & Hackett, 2022; McCrone & Osei‐Boateng, 2024). Additionally, the increase in hemoglobin concentration ([Hb]) is initially driven by a fluid redistribution resulting in plasma volume reduction and subsequently by enhanced renal erythropoietin release, which accelerates erythrocyte production (Siebenmann et al., 2024).

On the other hand, adaptation refers to populations genetically—and consequently physiologically—adapted to high altitudes, such as Andeans, Tibetans, and Ethiopians, who exhibit distinct characteristics. Andeans show physiological erythrocytosis with high hemoglobin (Hb) levels and low oxygen saturation (around 85%). Tibetans and Ethiopians, however, maintain hemoglobin levels like sea‐level populations, though Tibetans have a slightly lower average oxygen saturation (around 88%) than Ethiopians (Moore, 2017). Elevated Hb in Andean populations may primarily reflect long‐term acclimatization to high altitude rather than a genetic adaptation. This interpretation is supported by evidence showing that lowlanders residing at comparable altitudes for several months attain Hb values similar to those observed in Andeans, and that [Hb] levels in Andeans decrease toward normal ranges following descent to low altitude. Also, the elevated hematocrit observed in Andean populations increases blood viscosity and, consequently, vascular resistance, particularly within the pulmonary circulation. This haemodynamic alteration contributes to a substantial health burden and plays a central role in the pathophysiology of chronic mountain sickness (Siebenmann et al., 2024). These adapted groups also show reduced PAP via nitric oxide that comes from the airway wall to the pulmonary lumen (Moore, 2017). On the other hand, Andeans have mild right ventricular hypertrophy as a consequence of the elevated PAP and so in high altitude natives their Left ventricle (LV) and End diastolic Volume (EDV) are lower (Williams et al., 2022). Andean newborns (genetically adapted) do not have such lower birth weights compared to “physiologically” adapted populations (Beall et al., 2002). Tibetans have less prevalence of chronic mountain sickness compared to other ancestry groups also living in high altitudes (Moore, 2017; Niermeyer et al., 2015; Zidan et al., 2025).

## 3 EFFECTS OF FACE MASKS IN HEALTHY ADULTS

**FIGURE 1:**
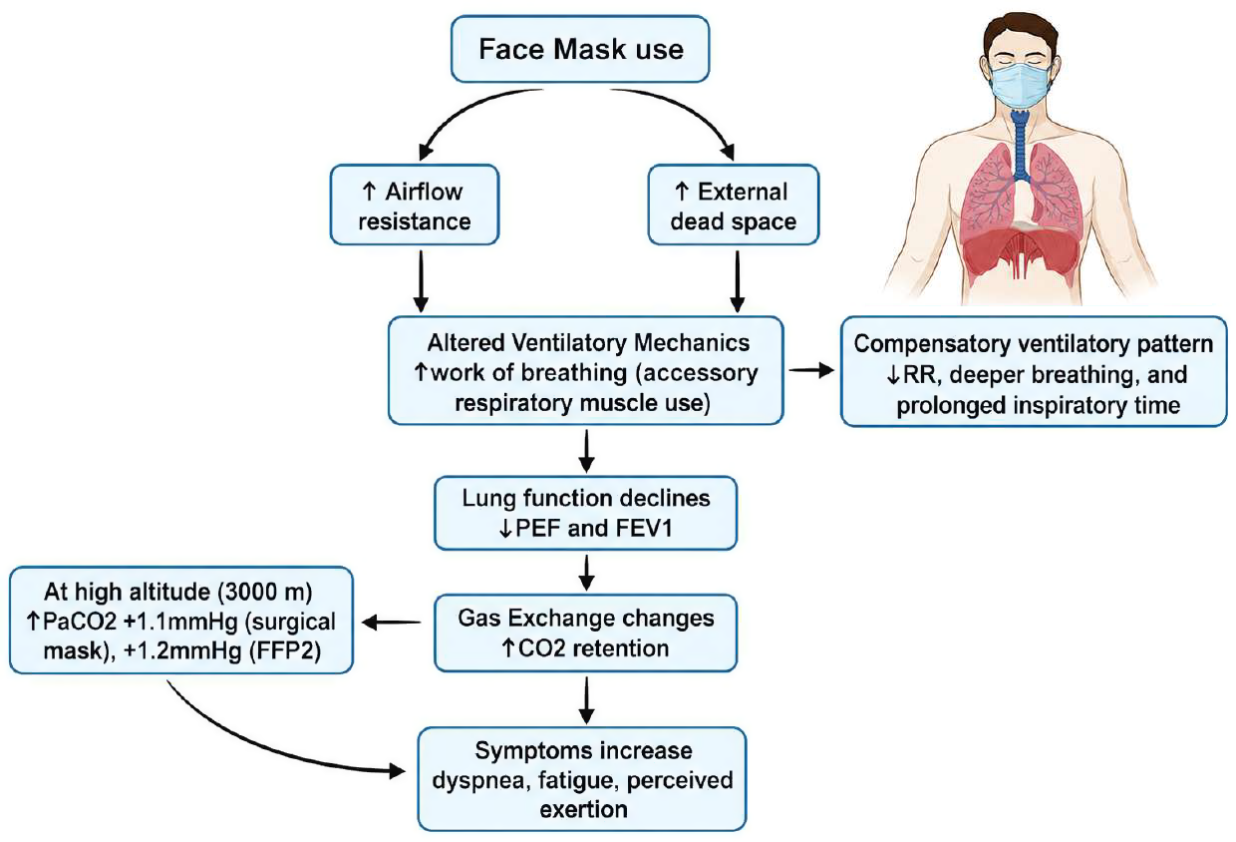
Effects of face mask in healthy adults.

The use of facial protective devices, particularly high‐efficiency respirators such as FFP2/N95 masks, induces alteration in ventilatory mechanics by increasing extrinsic airflow resistance (Figure 1) as a face mask acts as a supplementary physical barrier exerting external inspiratory resistance derived from its multilayered composition and the specific properties of the textile materials employed (Zheng et al., 2023), which translates into a substantial reduction in dynamic lung parameters, notably including drops of up to 21.3% in peak expiratory flow (PEF) and correlative decreases in forced expiratory volume in 1 s (FEV 1) (Figure 1). This additional resistive load elevates the work of breathing for the accessory musculature, limiting total ventilatory capacity and necessitating a compensatory adaptation of the ventilatory pattern characterized by a decrease in respiratory frequency and a significant prolongation of inspiratory time to overcome the impedance imposed by the filtering material (Figure 1). This transition toward a deeper, slower breathing regimen represents a homeostatic response to subjective dyspnea and flow restriction, evidencing a compromise in gas exchange efficiency under conditions of increased mechanical load (Fikenzer et al., 2020).

At sea level, a systematic review and meta‐analysis of 43 trials found that wearing surgical, cloth, or FFP2/N95 masks during exercise compared to no mask use moderately impairs gas exchange by reducing end‐tidal oxygen partial pressure (PetO_2_), while increasing end‐tidal carbon dioxide partial pressure (PetCO_2_); however, SaO_2_ was minimally impaired, and HR remained unchanged; perceived exertion, dyspnea, and fatigue increased notably (Figure 1) (Zheng et al., 2023). The reported reduction in submaximal oxygen consumption (VO_2_) has been criticized in Fikenzer's study (2020) because there was no difference in arterial CO_2_ pressure between FFP2/N95 face mask and unmasked conditions, and the PaO_2_ and pH were similar, so these results have been attributed to technical underestimation of minute ventilation (Hopkins et al., 2020). The increasing PetCO_2_ occurs because the use of a face mask creates a sequestered volume of air between the facial interface and the filtering media, resulting in a significant expansion of the external dead space. Consequently, upon exhalation, a fraction of the expired carbon dioxide is retained within this space (Figure 1) and subsequently re‐entrained during the following inspiratory phase, a phenomenon formally recognized as CO_2_ rebreathing (Figure 1) (Zheng et al., 2023).

At a simulated altitude of 3000 m, a randomized controlled crossover trial examined the clinical and physiological effects of surgical and FFP2 mask use. Eight healthy volunteers underwent masked and unmasked sessions in a normoxic and hypobaric hypoxia condition during rest and moderate exercise. Mask use slightly increased PaCO_2_ (+1.1 mmHg with surgical mask and +1.2 mmHg with FFP2 mask) (Figure 1). However, no significant changes in SaO_2_, PaO_2_, HR, or RR during either rest or exercise were reported. The minor changes in blood gases observed with mask use suggest a marginal decline in alveolar ventilation, this is explained for the expansion of external dead space and the absence of the expected compensatory ventilatory increase. There is an attenuated ventilatory response to dead space by the slower inspiratory and expiratory flow patterns reported in young and middle‐aged adults, likely secondary to the increased external airway resistance (Figure 1). Finally, mask use was associated with increased dyspnea and discomfort, cognitive performance remained unaffected (Vinetti et al., 2023).

## 4 COPD PREVALENCE AT HIGH ALTITUDE

Increasing evidence suggests that COPD prevalence decreases significantly in high altitude settings (Guo et al., 2020; Wen et al., 2025). A large cross‐sectional survey (*n* = 4967) of residents living at 2100–4700 m found a progressively significant decrease in COPD prevalence with increasing altitudes, reporting 12.1%, 6.9% and 5.4% at 2100–3000 m, 3000–4000 m, and >4000 m, respectively (*p* = 0.0001) (Guo et al., 2020). Another study with 11,095 patients showed that COPD prevalence among high‐risk individuals declined from 26.4% to 17.9% in low (<1500 m) to high (>2500 m) altitudes, respectively (*p* < 0.05) (Wen et al., 2025). Conversely, the PREPOCOL‐PLATINO‐BOLD‐EPI‐SCAN multivariate analysis found no significant difference in COPD prevalence between populations living above and below 1500 m after adjusting for age, sex, body mass index, level of education, smoking status, history of occupational exposure to dust, and former tuberculosis. However, COPD was undiagnosed in high altitudes due to fewer respiratory symptoms (Horner et al., 2017).

An analysis of the SPIROMICS trial showed a correlation between COPD patients living above 1219 m and mortality (hazard ratio: 1.25) compared to those living at lower elevations (<305 m). However, adjustment for air quality attenuated this association (Suri et al., 2024). High altitudes could alter disease pathophysiology and clinical manifestations, which may also apply to COPD.

## 5 EFFECTS OF FACE MASKS IN COPD PATIENTS

**FIGURE 2:**
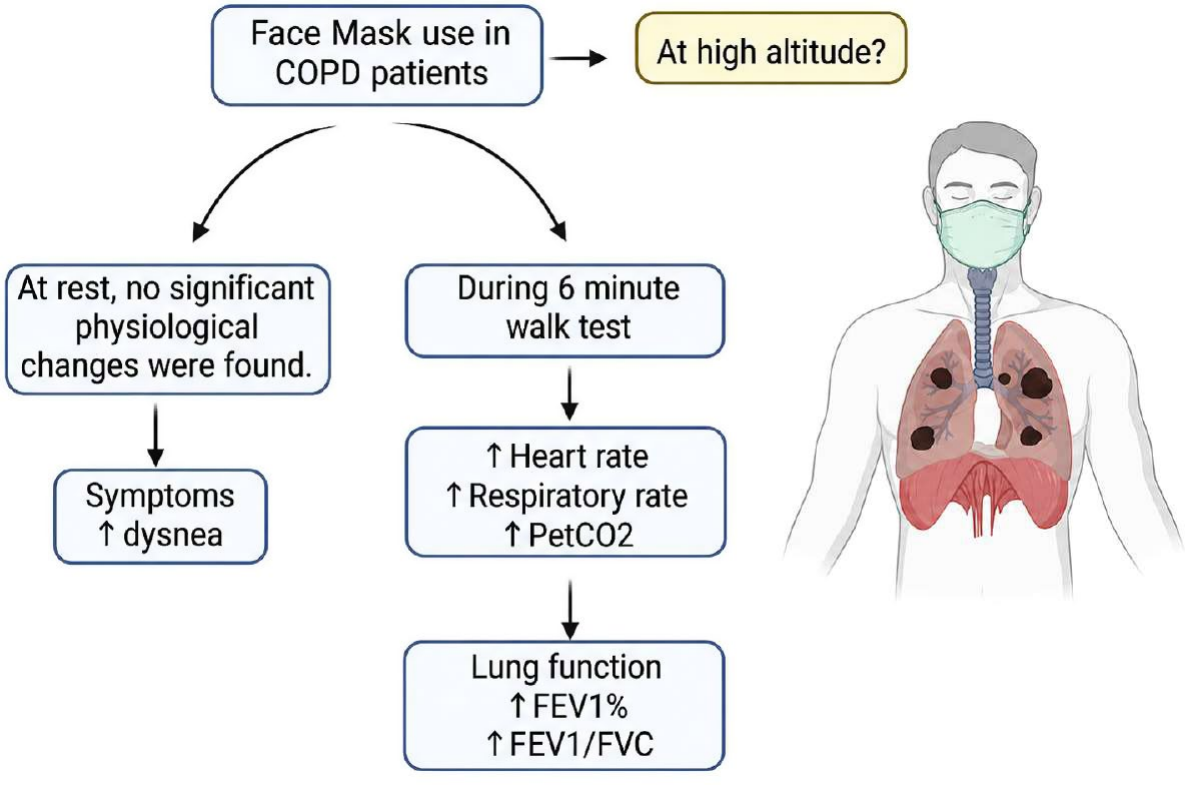
Effects of face mask in COPD patients.

Few studies have evaluated the *safety* of surgical mask use in COPD. No significant changes in blood pressure (BP), SaO_2_, HR, RR were found comparing surgical to N95 masks during rest and at a 6‐min walk test, despite the increased dyspnea (Figure 2) (Fan et al., 2025; Just et al., 2021; Samannan et al., 2020). Given the small sample sizes and brief mask‐wearing intervals, it is important to validate these findings in high altitude settings (Horner et al., 2017). During a 6‐min walk test, wearing an N95 face mask increased HR, RR, and PetCO_2_ (Figure 2) compared with those not wearing face masks (88–92 bpm; 23.3–25.7 breaths/min; 34–35.5 mmHg, respectively) but SpO_2_ declined slightly (93.8%–93%) (Kyung et al., 2020). On the other hand, mask use can increase FEV 1% and FEV1/FVC (Figure 2) due partially to their anti‐haze effect, minimizing air pollution (Chen et al., 2022). At higher altitudes, the reduced PIO_2_ may exacerbate the cardiorespiratory strain associated with N95 mask use, increasing the likelihood of oxygen desaturation and hypercapnia in COPD patients, especially in those with advanced disease. The available evidence on the effects of mask use in patients with COPD is limited and heterogeneous; short‐term studies show generally modest physiological changes, and data on long‐term outcomes such as exacerbations or hospitalizations are lacking.

In asthma, an important associated condition, the effects of face mask use in high altitude populations are uncertain. At sea level, its use in adolescents with well‐controlled asthma does not significantly affect SaO_2_ compared to poorly controlled asthma. On the other hand, warm and humid air retained within the mask may reduce the risk of exercise‐induced asthma attacks (Wen et al., 2025). In low‐oxygen environments, such as high‐altitude settings, the effects of surgical mask use on the cardiorespiratory system in individuals with asthma remain unclear. Therefore, further studies are needed.

## 6 CONCLUSIONS

Although available evidence suggests only limited short‐term physiological effects of face mask use, its real health impact in patients with COPD, particularly during prolonged use and under high‐altitude conditions, remains largely unknown. Further prospective studies are needed to better define the acute and chronic consequences in this vulnerable population.

## AUTHOR CONTRIBUTIONS

**Javier Josue Flores‐Mamani:** Conceptualization; investigation. **Andree Ingala‐Tapia:** Investigation. **Armin Alvaro Quispe‐Cornejo:** Investigation; supervision.

## FUNDING INFORMATION

This work did not receive any specific grant from funding agencies in the public, commercial, or not‐for‐profit sectors.

## CONFLICT OF INTEREST STATEMENT

No conflicts of interest, financial or otherwise, are declared by the authors.

## ETHICS STATEMENT

Ethical approval was not required because this study was based exclusively on publish.

## Data Availability

Not applicable.

## References

[phy271010-bib-0001] Baylis, C. , & Till, C. (2009). Interpretation of arterial blood gases. Surgery (Oxford), 27(11), 470–474. 10.1016/j.mpsur.2009.09.006

[phy271010-bib-0002] Beall, C. M. , Decker, M. J. , Brittenham, G. M. , Kushner, I. , Gebremedhin, A. , & Strohl, K. P. (2002). An Ethiopian pattern of human adaptation to high‐altitude hypoxia. Proceedings of the National Academy of Sciences of the United States of America, 99(26), 17215–17218. 10.1073/pnas.252649199 12471159 PMC139295

[phy271010-bib-0003] CDC . (2025). High‐Altitude Travel and Altitude Illness. In Yellow Book. Centers for Disease Control and Prevention. https://www.cdc.gov/yellow‐book/hcp/environmental‐hazards‐risks/high‐altitude‐travel‐and‐altitude‐illness.html 41818531

[phy271010-bib-0004] Chen, X. , Zhang, C. , Ibrahim, S. , Tao, S. , Xia, X. , Li, Y. , Li, C. , Yue, F. , Wang, X. , Bao, S. , & Fan, J. (2022). The impact of facemask on patients with COPD: A systematic review and meta‐analysis. Frontiers in Public Health, 10, 1027521. 10.3389/fpubh.2022.1027521 36466486 PMC9709116

[phy271010-bib-0005] Fan, J. , Feng, T. , Jiang, X. , Wei, C. , Zhang, X. , Li, C. , Yue, F. , Yang, H. , Bao, S. , & Chen, X. (2025). Assessing the impact of different types of masks on COPD patients: A randomised controlled trial. ERJ Open Research, 11(2), 00806‐2024. 10.1183/23120541.00806-2024 40129539 PMC11931569

[phy271010-bib-0006] Fikenzer, S. , Uhe, T. , Lavall, D. , Rudolph, U. , Falz, R. , Busse, M. , Hepp, P. , & Laufs, U. (2020). Effects of surgical and FFP2/N95 face masks on cardiopulmonary exercise capacity. Clinical Research in Cardiology, 109(12), 1522–1530. 10.1007/s00392-020-01704-y 32632523 PMC7338098

[phy271010-bib-0007] Grocott, M. P. W. , Martin, D. S. , Levett, D. Z. H. , McMorrow, R. , Windsor, J. , & Montgomery, H. E. (2009). Arterial blood gases and oxygen content in climbers on Mount Everest. New England Journal of Medicine, 360(2), 140–149. 10.1056/NEJMoa0801581 19129527

[phy271010-bib-0008] Guo, Y. , Xing, Z. , Shan, G. , Janssens, J. P. , Sun, T. , Chai, D. , Liu, W. , Wang, Y. , Ma, Y. , Tong, Y. , & Huang, Y. (2020). Prevalence and risk factors for COPD at high altitude: A large cross‐sectional survey of subjects living between 2,100–4,700 m above sea level. Frontiers in Medicine, 7, 581763. 10.3389/fmed.2020.581763 33344472 PMC7744817

[phy271010-bib-0010] Hopkins, S. R. , Stickland, M. K. , Schoene, R. B. , Swenson, E. R. , & Luks, A. M. (2020). Effects of surgical and FFP2/N95 face masks on cardiopulmonary exercise capacity: The numbers do not add up. Clinical Research in Cardiology, 109, 1605–1606. 10.1007/s00392-020-01748-0 33034744 PMC7688504

[phy271010-bib-0011] Horner, A. , Soriano, J. B. , Puhan, M. A. , Studnicka, M. , Kaiser, B. , Vanfleteren, L. E. G. W. , Gnatiuc, L. , Burney, P. , Miravitlles, M. , García‐Rio, F. , & Ancochea, J. (2017). Altitude and COPD prevalence: Analysis of the PREPOCOL‐PLATINO‐BOLD‐EPI‐SCAN study. Respiratory Research, 18(1), 162. 10.1186/s12931-017-0643-5 28835234 PMC5569455

[phy271010-bib-0012] Imray, C. , Booth, A. , Wright, A. , & Bradwell, A. (2011). Acute altitude illnesses. 10.1136/bmj.d4943 21844157

[phy271010-bib-0013] Just, I. A. , Schoenrath, F. , Passinger, P. , Stein, J. , Kemper, D. , Knosalla, C. , Falk, V. , & Knierim, J. (2021). Validity of the 6‐minute walk test in patients with end‐stage lung diseases wearing an oronasal surgical mask in times of the COVID‐19 pandemic. Respiration; International Review of Thoracic Diseases, 100(7), 594–599. 10.1159/000515606 33878758 PMC8089427

[phy271010-bib-0014] Kyung, S. Y. , Kim, Y. , Hwang, H. , Park, J. W. , & Jeong, S. H. (2020). Risks of N95 face mask use in subjects with COPD. Respiratory Care, 65(5), 658–664. 10.4187/respcare.06713 31992666

[phy271010-bib-0015] Li, H. , Yuan, K. , Sun, Y. K. , Zheng, Y. B. , Xu, Y. Y. , Su, S. Z. , Zhang, Y. X. , Zhong, Y. , Wang, Y. J. , Tian, S. S. , & Gong, Y. M. (2022). Efficacy and practice of facemask use in general population: A systematic review and meta‐analysis. Translational Psychiatry, 12(1), 1–15. 10.1038/s41398-022-01814-3 35105851 PMC8804079

[phy271010-bib-0016] Luks, A. M. , & Hackett, P. H. (2022). Medical conditions and high‐altitude travel. New England Journal of Medicine, 386(4), 364–373. 10.1056/NEJMra2104829 35081281

[phy271010-bib-0017] McCrone, J. C. , & Osei‐Boateng, C. (2024). Haemoconcentrating on cerebral blood flow regulation during early acclimatization: Does altitude severity matter? Journal of Physiology, 602(21), 5721–5722. 10.1113/JP286928 39087848

[phy271010-bib-0018] Moore, L. G. (2017). Measuring high‐altitude adaptation. Journal of Applied Physiology, 123(5), 1371–1385. 10.1152/japplphysiol.00321.2017 28860167 PMC5792094

[phy271010-bib-0020] Niermeyer, S. , Andrade‐M, M. P. , Vargas, E. , & Moore, L. G. (2015). Neonatal oxygenation, pulmonary hypertension, and evolutionary adaptation to high altitude (2013 Grover conference series). Pulmonary Circulation, 5(1), 48–62. 10.1086/679719 25992270 PMC4405714

[phy271010-bib-0021] Samannan, R. , Holt, G. , Calderon‐Candelario, R. , Mirsaeidi, M. , & Campos, M. (2020). Effect of face masks on gas exchange in healthy persons and patients with chronic obstructive pulmonary disease. Annals of the American Thoracic Society, 18(3), 541–544. 10.1513/AnnalsATS.202007-812RL PMC7919152 33003954

[phy271010-bib-0022] Siebenmann, C. , Roche, J. , Schlittler, M. , Simpson, L. L. , & Stembridge, M. (2024). Regulation of haemoglobin concentration at high altitude. The Journal of Physiology, 602(21), 5587–5600. 10.1113/JP284578 38051656

[phy271010-bib-0023] Suri, R. , Markovic, D. , Woo, H. , Arjomandi, M. , Barr, R. G. , Bowler, R. P. , Criner, G. , Curtis, J. L. , Dransfield, M. T. , Drummond, M. B. , & Fortis, S. (2024). The effect of chronic altitude exposure on chronic obstructive pulmonary disease outcomes in the SPIROMICS cohort: An observational cohort study. American Journal of Respiratory and Critical Care Medicine, 210, 1210–1218. 10.1164/rccm.202310-1965OC 38507607 PMC11568439

[phy271010-bib-0024] Tremblay, J. C. , & Ainslie, P. N. (2021). Global and country‐level estimates of human population at high altitude. Proceedings of the National Academy of Sciences of the United States of America, 118(18), e2102463118. 10.1073/pnas.2102463118 33903258 PMC8106311

[phy271010-bib-0025] Vinetti, G. , Micarelli, A. , Falla, M. , Randi, A. , Dal Cappello, T. , Gatterer, H. , Brugger, H. , Strapazzon, G. , & Rauch, S. (2023). Surgical masks and filtering facepiece class 2 respirators (FFP2) have no major physiological effects at rest and during moderate exercise at 3000‐m altitude: A randomised controlled trial. Journal of Travel Medicine, 30(5), taad031. 10.1093/jtm/taad031 36881665 PMC10481409

[phy271010-bib-0026] Wen, G. , Meng, J. , Wang, H. , Peng, P. , Xu, Y. , Wang, R. , Yan, Z. , Du, B. , Wen, A. , Luo, G. , & Cui, W. (2025). Prevalence of chronic obstructive pulmonary disease in high‐risk populations at low, intermediate, high altitudes: A population based cross‐sectional study in Yunnan Province, China. BMC Pulmonary Medicine, 25(1), 124. 10.1186/s12890-025-03565-5 40102845 PMC11916964

[phy271010-bib-0027] West, J. , Schoene, R. , Luks, A. , & Milledge, J. (2012). High Altitude Medicine and Physiology 5E (5th ed., p. 584). CRC Press. 10.1201/b13633

[phy271010-bib-0028] Williams, A. M. , Levine, B. D. , & Stembridge, M. (2022). A change of heart: Mechanisms of cardiac adaptation to acute and chronic hypoxia. The Journal of Physiology, 600(18), 4089–4104. 10.1113/JP281724 35930370 PMC9544656

[phy271010-bib-0029] World Health Organization . (2024). The top 10 causes of death. https://www.who.int/news‐room/fact‐sheets/detail/the‐top‐10‐causes‐of‐death

[phy271010-bib-0030] World Health Organization . (2026). Personal‐level actions to reduce air pollution exposure in the WHO European Region. https://iris.who.int/items/98de30fc‐e1d2‐4351‐acfc‐d49f62457145

[phy271010-bib-0031] Zheng, C. , Poon, E. T. C. , Wan, K. , Dai, Z. , & Wong, S. H. S. (2023). Effects of wearing a mask during exercise on physiological and psychological outcomes in healthy individuals: A systematic review and meta‐analysis. Sports Medicine, 53(1), 125–150. 10.1007/s40279-022-01746-4 36001290 PMC9400006

[phy271010-bib-0032] Zidan, B. M. R. M. , Zehravi, M. , Kareemulla, S. , Madhuri, K. S. D. , Gupta, J. K. , Kumar, V. V. , Khan, J. , Alkhathami, A. G. , Osman, H. , Khandaker, M. U. , & Emran, T. B. (2025). High‐altitude physiology: Understanding molecular, pharmacological and clinical insights. Pathology‐Research and Practice, 272, 156080. 10.1016/j.prp.2025.156080 40516140

